# A Turkish mobile application for single-word auditory comprehension in aphasia: development and preclinical evaluation

**DOI:** 10.3389/fneur.2026.1830403

**Published:** 2026-10-28

**Authors:** Rabia Aygün, Suna Tokgöz-Yılmaz

**Affiliations:** 1 Department of Speech and Language Therapy, Faculty of Health Sciences, Istanbul Medeniyet University, Istanbul, Türkiye; 2 Audiology, Balance and Speech Disorders Unit, Department of Otorhinolaryngology, School of Medicine, Ankara University, Ankara, Türkiye

**Keywords:** aphasia, auditory comprehension, mobile application, speech and language therapy, telepractice

## Abstract

**Purpose:**

Aphasia frequently impairs auditory comprehension and limits everyday communication. This study describes the design and development of a Turkish-language mobile application targeting single-word auditory comprehension and reports initial performance and usability findings from healthy adults to inform subsequent clinical testing.

**Materials and methods:**

In the development phase, 84 healthy adults (49 women, 35 men, aged 19–31 years) evaluated visual stimuli using name agreement and image agreement procedures. After revising items with low agreement, a mobile application comprising hierarchically organized single-word auditory comprehension tasks across 12 semantic categories was developed. The finalized application was administered to 40 healthy participants (20 women, 20 men) aged 18–80 years. Accuracy, response time, and usability outcomes were analyzed by age, education level, and mobile device use skills.

**Results:**

Significant group differences were observed. Participants aged ≥60 years showed lower accuracy and longer response time than the younger groups (*p* < 0.05). Higher education levels and ‘Good and Above’ mobile device use skills were generally associated with higher accuracy and shorter response time (*p* < 0.05). Daily mobile device use showed no consistent associations with performance (*p* > 0.05). Accuracy exceeded 90% across all categories. The application was rated ‘definitely usable’ on the Mobile Application Usability Scale.

**Conclusion:**

This study presents the design and preclinical evaluation of a Turkish-language mobile application targeting single-word auditory comprehension. The application demonstrated high usability and strong overall task performance in healthy adults, providing initial support for further evaluation in clinical populations. Future studies should investigate its feasibility, clinical relevance, and therapeutic potential in individuals with aphasia.

## Introduction

Aphasia is an acquired language disorder caused by brain damage, characterized by impairments in expressive language, auditory comprehension, repetition, naming, reading, and writing (1). It most often results from stroke, traumatic brain injury, or tumors, and affects millions worldwide (2). Beyond impairing linguistic ability, aphasia profoundly disrupts social engagement, psychological wellbeing, and overall quality of life (3). Rehabilitation is therefore a complex, multidimensional process, and strengthening auditory comprehension is especially critical, as accurate perception and processing of spoken input are prerequisites for effective language use.

Auditory comprehension deficits are particularly common in non-fluent aphasia. Many individuals struggle to understand spoken language to varying degrees, making it one of the most disabling symptoms. Difficulties in perceiving and interpreting auditory information interrupt conversational flow and limit participation in social interaction (4). Despite this significant impact, auditory comprehension has received less research and clinical attention in aphasia compared to other language modalities, such as naming and expressive language (5). This gap is particularly evident in the Turkish context, where structured intervention tools specifically targeting auditory comprehension remain scarce.

Interventions that target auditory comprehension are a key component in improving overall communication abilities. Given the substantial impact of aphasia, individuals are often enrolled in speech and language therapy to address language deficits. A substantial body of research supports the sustained, positive clinical outcomes of such interventions (6). In a comprehensive review, Brady et al. (3) found that speech and language therapy, when added to standard care for individuals with post-stroke aphasia, significantly improves functional communication, reading, writing, and expressive language. Moreover, they reported strong evidence that higher therapy intensity (e.g., increased weekly session frequency) or longer treatment duration (e.g., greater total number of weeks) yields superior outcomes.

Despite this, public healthcare systems often cannot provide the required extended access, leaving many patients and families without sufficient long-term or intensive support (7). Conventional rehabilitation usually involves face-to-face sessions, delivered one-on-one by speech and language therapists and tailored to individual needs (3, 7). Yet the persistent gap between the demand for intensive therapy and service limitations—including cost, transport barriers, geographic distance, and therapist shortages—makes it difficult to deliver treatment at the required frequency and duration (6, 8). These constraints have prompted a sustained search for complementary delivery models, with clinicians increasingly encouraged to integrate technology into both assessment and treatment (9, 10). In a review of the telerehabilitation literature in aphasia, Cacciante et al. (11) concluded that remotely delivered therapy can achieve outcomes comparable to in-person care while expanding access for underserved populations, supporting its role as a complement to—rather than a replacement for—clinician-led treatment.

Among technology-supported approaches, mobile applications have become one of the most promising tools in aphasia rehabilitation. Smartphones and tablets are portable, easy to personalize, and already integrated into daily life, making them practical for healthcare use (12). They are now widely used in speech and language therapy, both to support clinician-led sessions and to allow patients to practice on their own (13, 14). This capacity for independent practice is particularly relevant given the growing emphasis on self-management in aphasia, where patients take a more active role alongside therapists and caregivers (15). Recent systematic reviews show that mobile- and tablet-based interventions can improve overall language skills, although gains in naming and everyday communication are smaller and the evidence remains limited by small samples and mixed study quality (16–18). Together, these features make purpose-built mobile applications a valuable means of increasing therapy frequency and supporting independent practice—provided they are carefully matched to the target language, the specific linguistic domain, and the intended population.

Such matching, however, remains uneven across languages. A range of computer-based and mobile applications has been developed for aphasia therapy, but most target English or other widely spoken languages (16, 19–25). Because aphasia is inherently language-specific, these tools cannot simply be transferred to other linguistic and cultural contexts. In Turkish, existing applications have focused mainly on naming (26), leaving other clinically important language domains, particularly auditory comprehension, largely unaddressed.

A second key consideration in developing such tools is the end user. Beyond language and clinical content, factors such as age, educational background, and technological literacy strongly influence usability and engagement, and applications that overlook them risk reducing both therapeutic effectiveness and patient motivation. This is especially relevant in aphasia, as most affected individuals are older adults whose digital experience varies widely and whose language impairment further constrains their interaction with conventional interfaces. Designs that account for these characteristics—through simplified navigation, clear visual and auditory cues, and minimal reliance on text—are therefore central, rather than secondary, to therapeutic effectiveness.

Accordingly, the present study aimed to (1) describe the design and development of a Turkish-language mobile application targeting single-word auditory comprehension in aphasia, and (2) report initial performance and usability findings from healthy adults as a preclinical evaluation step intended to inform subsequent clinical studies in individuals with aphasia and to guide further refinement of the application before therapeutic implementation. To our knowledge, this is the first Turkish-language mobile application specifically developed to target auditory comprehension in aphasia.

## Materials and methods

This study protocol was reviewed and approved by an institutional human research ethics committee (approval number: [İ03-118-22]).

### Participants

During development of the mobile application, 84 participants (49 women, 35 men, mean age = 21.86 ± 2.18 years) evaluated the images using name-image agreement procedures. Based on the findings from this phase, the prototype mobile application was subsequently evaluated by 40 healthy individuals (20 women, 20 men) in three age groups: 18–39 years (*n* = 15), 40–59 years (*n* = 12), and ≥ 60 years (*n* = 13).

Inclusion criteria were pure-tone hearing thresholds ≤20 dB HL at 500, 1000, 2000, and 4,000 Hz; normal otoscopy and immittance; Mini-Mental State Examination (MMSE) score ≥24; literacy; and general skills for operating the application. Exclusion criteria included major visual impairment, cognitive or physical limitations, and insufficient mobile device skills. Insufficient mobile device skills were operationally defined as self-reported difficulty in performing basic smartphone functions required for the task (e.g., navigating applications, selecting images via touchscreen, and following on-screen instructions).

### Data collection tools

#### Demographic information form

After being informed about the study, participants completed a demographic information form that included age, sex, education level, and occupation, as well as questions about technology usage. The form was completed prior to testing. Daily mobile device use was reported by participants in three categories: 0–2 h, 2–4 h, and 5 h or more. Mobile device use skills were self-rated by participants using a single item with four response options (‘Very good,’ ‘Good,’ ‘Moderate,’ ‘Poor’), with reference to common smartphone tasks such as opening applications, navigating between screens, and selecting options via touchscreen. For analysis, responses were dichotomized into ‘Good and Above’ (Good, Very good) and ‘Moderate and Below’ (Moderate, Poor) to ensure adequate subgroup sample sizes.

#### Hearing assessment

Because the mobile application used auditory stimuli and targeted auditory comprehension, participants were required to have normal hearing. Pure-tone thresholds were measured using an AC-40 clinical audiometer (Interacoustics A/S, Denmark) with Telephonics TDH 39P headphones. Tympanometry and acoustic reflexes were assessed with a GSI TympStar Pro immittance meter (Grason-Stadler, USA). Participants with mean thresholds ≤20 dB HL at 500–4000 Hz and normal immittance were included (27).

#### Mini-Mental State Examination (MMSE)

The MMSE, developed by Folstein et al. (28), is widely used as a cognitive screening tool. Administered in approximately 10 min, it comprises 11 items assessing registration, recall, language, attention/calculation, and orientation, with a maximum score of 30. Originally designed for clinicians, it is now also used to determine eligibility for clinical research. The Turkish validity and reliability study was conducted by Güngen et al. (29). In this study, participants’ cognition was evaluated with the MMSE. Individuals scoring < 24 were excluded.

#### Mobile Application Usability Scale (MAUS)

According to Venkatesh and Ramesh (30), usability refers to the extent to which an application performs tasks effectively, efficiently, and with user satisfaction. Several scales exist for this purpose (31–34). The MAUS, developed by Hoehle et al. (35) based on Microsoft’s mobile usability guidelines, was adapted to Turkish by Güler (36).

The MAUS includes 10 factors—Aesthetic, Color, Control, Entry, Fingertip, Font, Gestalt, Hierarchy, Animation, and Transition. Each factor has 4 items rated on a 7-point Likert scale (1 = “strongly disagree” to 7 = “strongly agree”), totaling 40 items. Factor scores are the sum of their items; the total MAUS score is the sum of all factors. After using the application, all participants completed the MAUS. In addition, opinions and suggestions were collected through an open-ended question.

### Method

In their review, Griffel et al. (37) examined the methodologies of mobile applications developed for individuals with aphasia and proposed a model method consisting of three stages: theoretical preparation, application development, and evaluation. Based on this model, our study also followed these three stages.

#### Theoretical preparation

In this stage, the objectives for developing the mobile application, the characteristics of the therapy, therapeutic requirements, exercises, and materials (visual, written, auditory) were determined. A feasibility check was conducted with a software engineer.

Exercises to improve auditory comprehension were designed hierarchically. Materials included words categorized using Turkish frequency lists, images from paid and free stock sources, and audio recorded with a professional voice actor. The suitability of words and images was evaluated using multiple methods.

##### Selection and evaluation of words and images

Word selection was supported by a linguist and focused on everyday life situations relevant to the application. The hierarchical design prioritized frequently used words, selected from Turkish frequency dictionaries based on written language corpora (38).

The psycholinguistic properties of images must be well-defined for language assessment and rehabilitation. The psycholinguistic properties (imageability, familiarity, and age of acquisition) of 1,128 words, previously analyzed (39), were considered in the application design. These words included concrete nouns, verbs, and abstract concepts. Of the 1,128 evaluated words, 778 with high imageability scores were selected. Corresponding images were obtained from paid and free stock image repositories. Images and their backgrounds were kept as simple as possible to accurately represent the intended noun or verb.

The dataset of 778 images was divided into two parts, which were then evaluated for name and image agreement. The evaluation involved 84 participants aged 19–31 years. For name agreement, participants briefly viewed images and wrote the first name that came to mind. Name agreement percentage and *H* value were calculated. The percentage was obtained by dividing the frequency of the most common name by the total number of responses. The *H* value reflected the number, proportions, and distribution of different names (40):


$$H=\sum_{i=1}^{k}p_{i}\log_{2}(1/p_{i})$$

Here, *k* refers to the number of names given, and pᵢ the proportion of participants giving each name. Higher *H* values indicate lower name agreement, whereas values close to zero or equal to zero reflect high name agreement. In the present study, images with *H* values of 1.0 or higher were revised or replaced to ensure adequate naming consistency.

For image agreement, participants were first presented with the most common name from the naming procedure and asked to visualize it for about 3 s. They were then shown the image and asked to rate how closely it matched their mental image. Agreement was rated on a five-point Likert scale, where 1 indicated low agreement (weak match) and 5 indicated high agreement.

#### Development of the mobile application

Following the acquisition and evaluation of the materials, a software engineer developed a prototype mobile application based on established criteria. This mobile application, designed for use by individuals with aphasia, their caregivers, or clinicians, aims to improve auditory comprehension skills. It consists of word comprehension tasks in which the target word is presented auditorily and the user selects the corresponding image by touch.

The application begins with an entry screen requesting personal details—name, gender, date of birth, education level, and occupation. It also includes basic questions about the type and symptoms of aphasia, to be completed by a family member or caregiver only during initial registration.

After registration, the user can select a category of words to work on from the category selection screen. Words in the application are organized into multiple semantic categories, which can be prioritized by the clinician, patient, or caregiver based on the patient’s needs. The application contains twelve categories: “Clothing,” “Foods and Drinks,” “Plants and Animals,” “Objects,” “Verbs,” “Adjectives,” “General Concepts,” “Body Parts,” “People,” “Occupations,” “Places,” and “Vehicles” (Figure 1). These categories were determined by reviewing studies on semantic fluency (41–46) and personally relevant vocabulary in aphasia (47).

**Figure 1:**
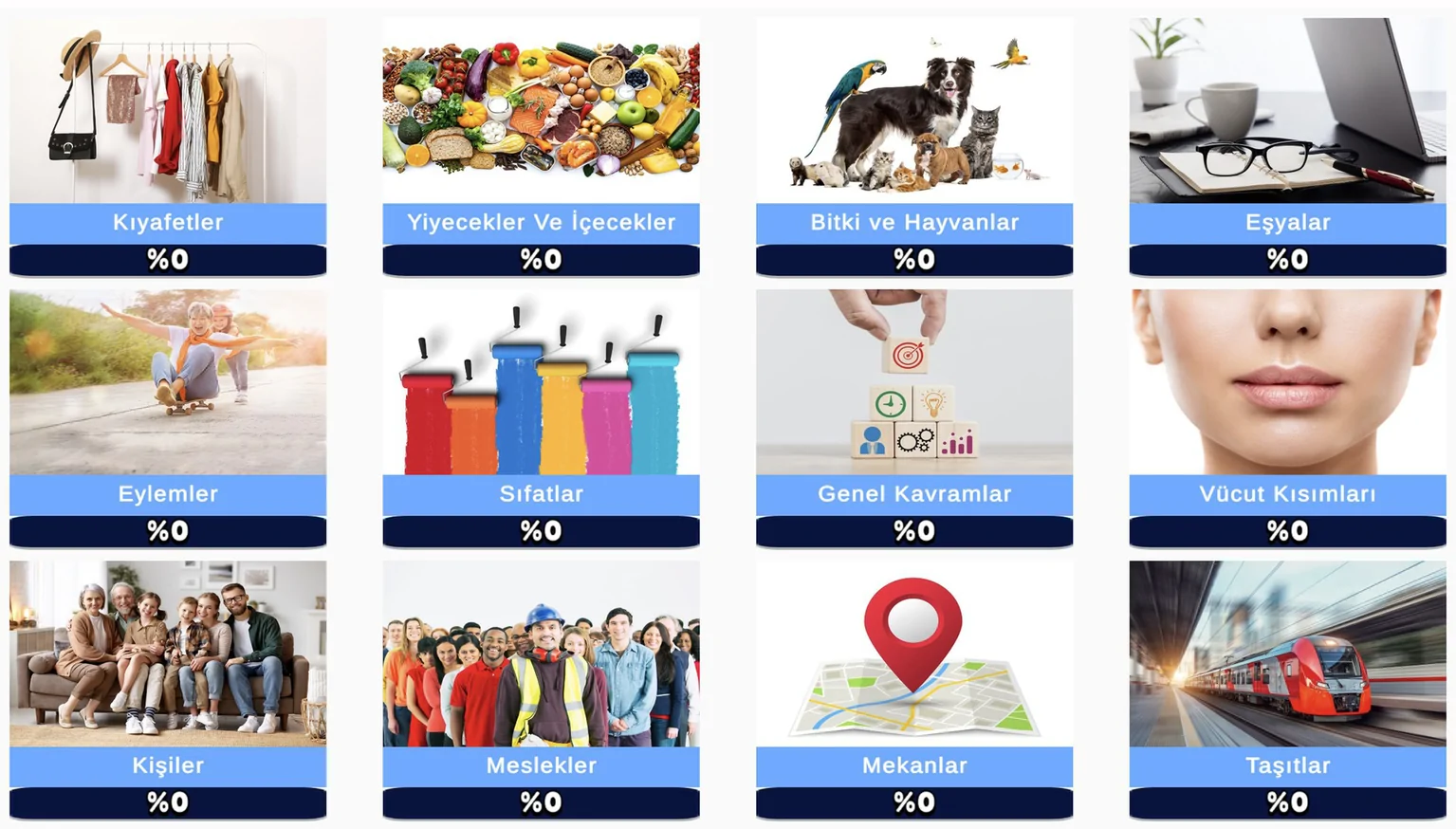
Categories screen of the mobile application. Images adapted with permission from iStock (https://www.istockphoto.com).

The categories are divided into sections, and the number of sections within each category varies. The “Clothing” category contains 4 sections, “Foods and Drinks” 13, “Plants and Animals” 6, “Objects” 15, “Verbs” 13, “Adjectives” 8, “General Concepts” 9, “Body Parts” 3, “People” 2, “Occupations” 6, “Places” 7, and “Vehicles” 2 sections.

A hierarchical order of difficulty was established among the sections. Hierarchical learning can be supportive in therapy for individuals with aphasia (48–52). For this purpose, sections within the categories were created from words grouped according to semantic similarity. The words in each section were then ordered based on their average familiarity, age of acquisition, and imageability values. Sections containing words with earlier acquisition ages and higher imageability and familiarity were presented first, while those with later acquisition ages and lower values were presented later. For example, in the “Foods and Drinks” category, Section 1 was designed to be the easiest, and Section 13 the most difficult. In this way, words encountered more frequently in daily life can be practiced earlier. Figure 2 shows the words and images from Sections 1 and 10 of the “Foods and Drinks” category, representing the easiest level (Section 1) and a more difficult level (Section 10).

**Figure 2:**
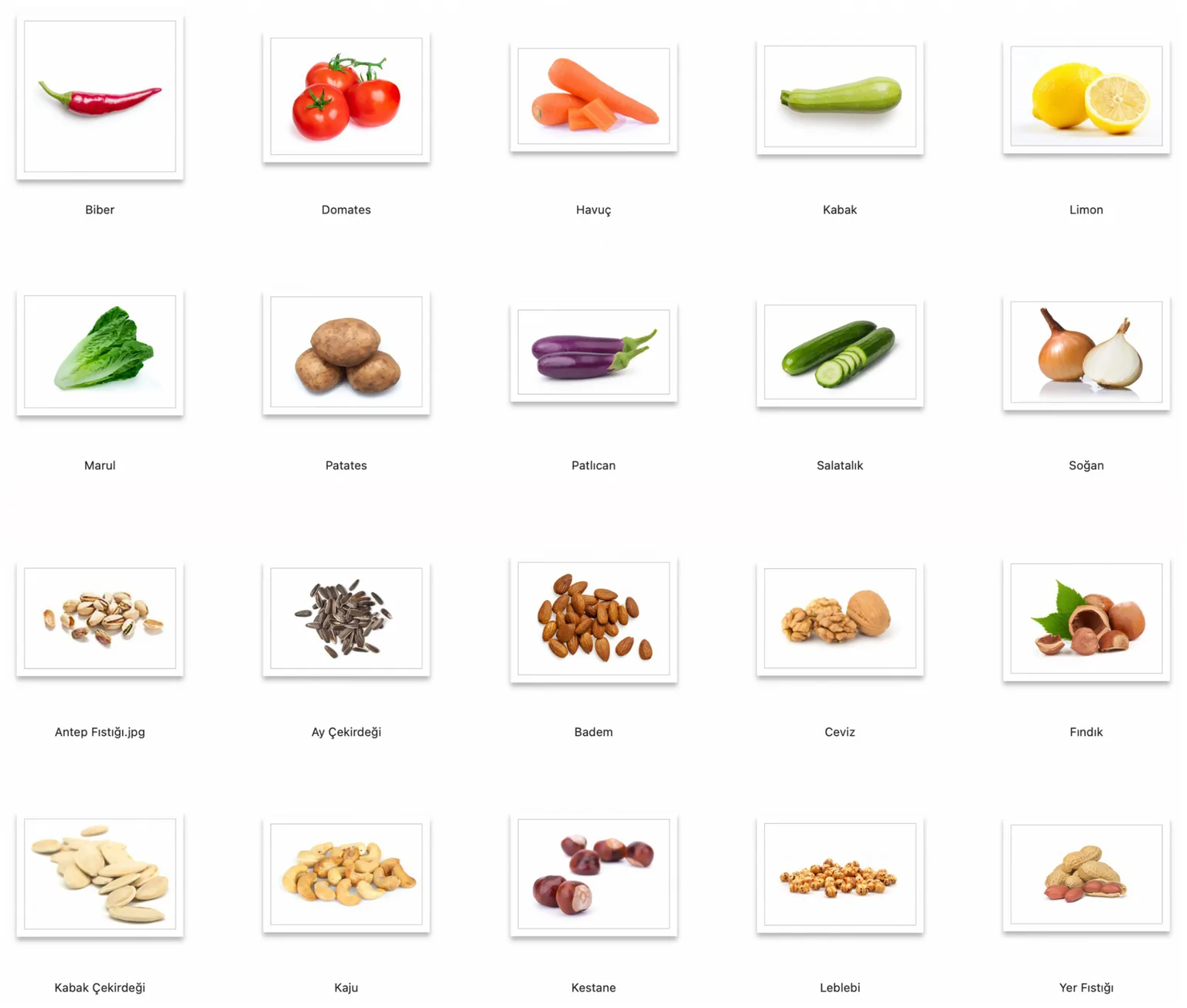
Words in sections 1 and 10 of the “Foods and Drinks” category. The top image shows the words from difficulty level 1, and the bottom image shows the words from difficulty level 10. Images adapted with permission from Envato Elements (https://elements.envato.com).

After the category and section are selected, the user is directed to the *Settings* screen to configure how the application will be used. Before starting, three parameters must be set: *Number of Options*, *Difficulty Level*, and *Word Repetition Count*. The *Number of Options* determines how many alternatives are presented for each spoken word stimulus; depending on the patient’s general health status, aphasia type, and severity, *2*,*4*, or *6* options can be chosen.

The *Difficulty Level* is defined according to whether the target word and the distractors belong to the same semantic category. The application includes three levels—*easy*, *medium*, and *hard*: at the easy level, none of the distractors share the target’s semantic category; at the medium level, 50% do; and at the hard level, all distractors come from the same category as the target.

The *Word Repetition Count* specifies how many times each word in a section is presented per run, allowing clinicians to adjust the amount of repeated auditory exposure according to therapeutic needs.

Once the settings are finalized, the task screen prompts the patient to tap the image corresponding to the word they hear. If the patient needs to hear the word again, tapping the “Speaker” icon replays the target word. When needed, tapping the “Cue” icon reveals the written form of the word, which remains visible until the item ends.

Distinct sounds are played for correct and incorrect selections. After each incorrect response, the number of distractors is reduced, and the word is presented again as an auditory stimulus. If the patient is still unable to respond correctly when only two options remain, the correct option is enlarged, shown alone on the screen, replayed, and displayed in written form (Figure 3).

**Figure 3:**
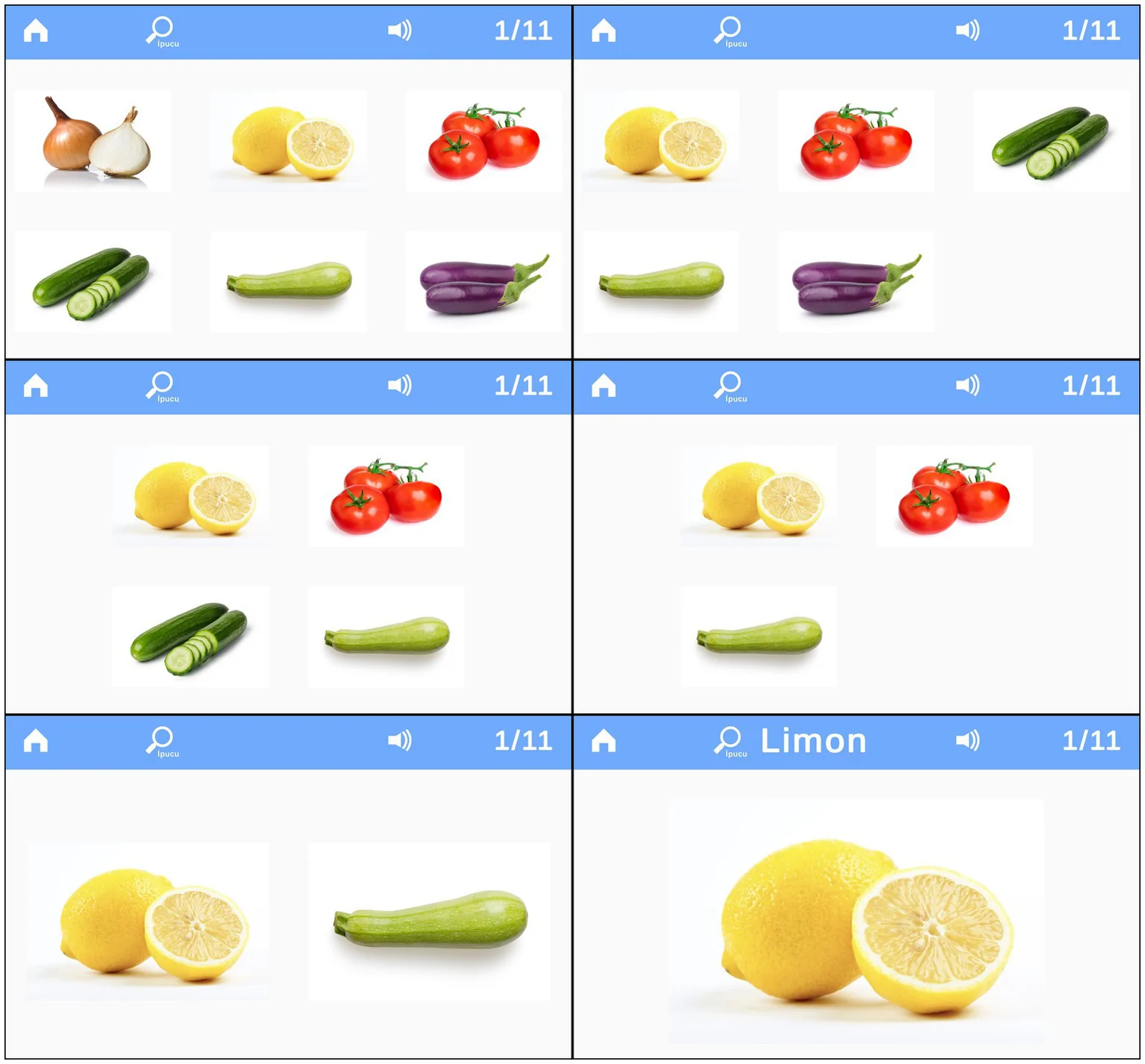
Illustration of the decreasing number of distractors following incorrect responses. The displayed example is for illustrative purposes only and does not represent actual participant data.

When a section is completed, the “Results” screen displays the settings used for that section, the total time spent, the average time per question (response time), and the percentage of correct responses (Figure 4). Based on data obtained from individuals with normal hearing, the threshold for section completion was set at a minimum of 90% correct answers. When this threshold is achieved, completion is visually indicated using specific icons. This feature facilitates the tracking of application usage and outcomes for patients, caregivers, and therapists.

**Figure 4:**
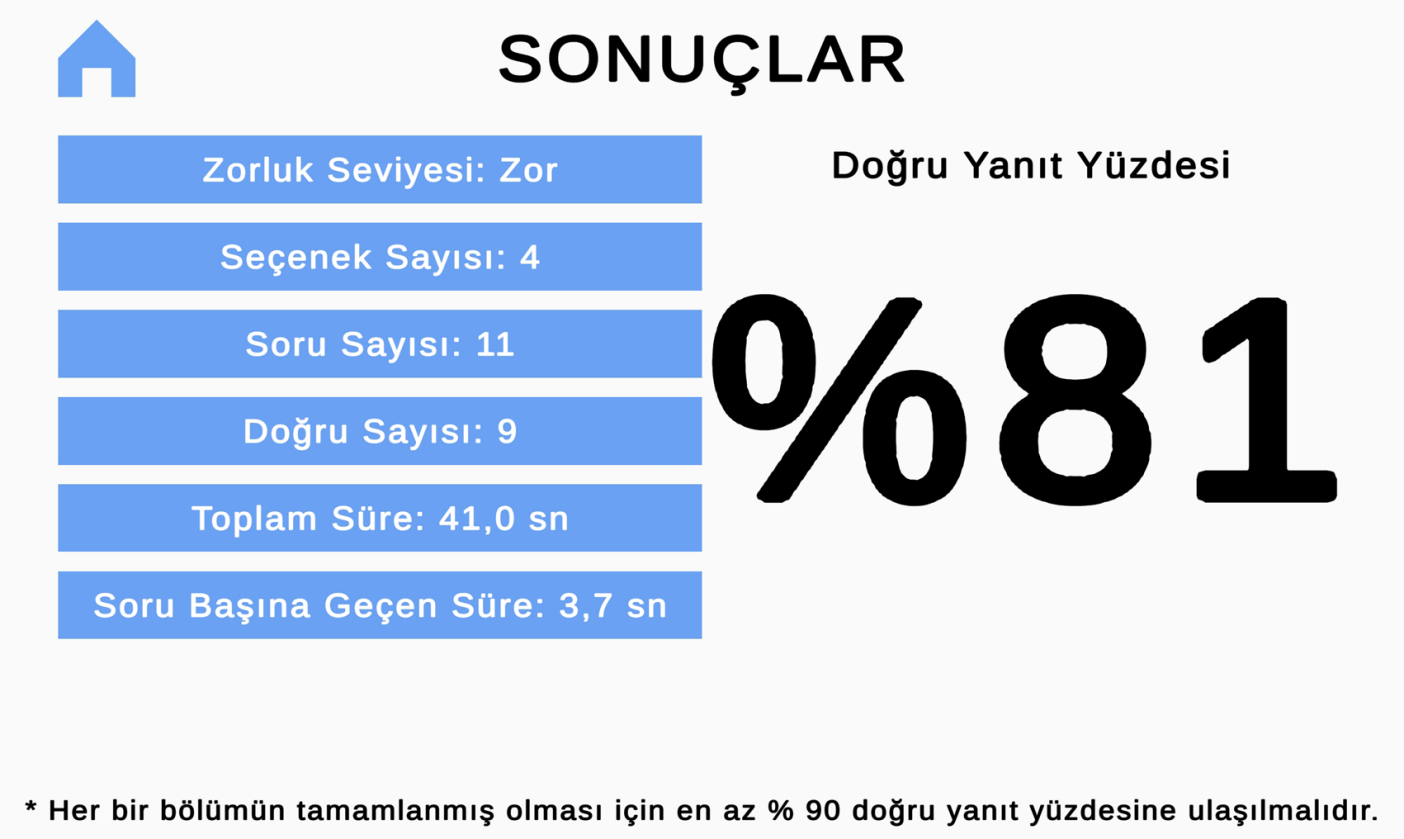
Results screen of the mobile application. The displayed values are for illustrative purposes only and do not represent actual participant data.

Progress bars were incorporated into categories and sections to display completion status, facilitate tracking, and enhance motivation (Figure 5). This allows users to resume their progress from the previous session and enables the identification of areas needing improvement, allowing training to be appropriately directed.

**Figure 5:**
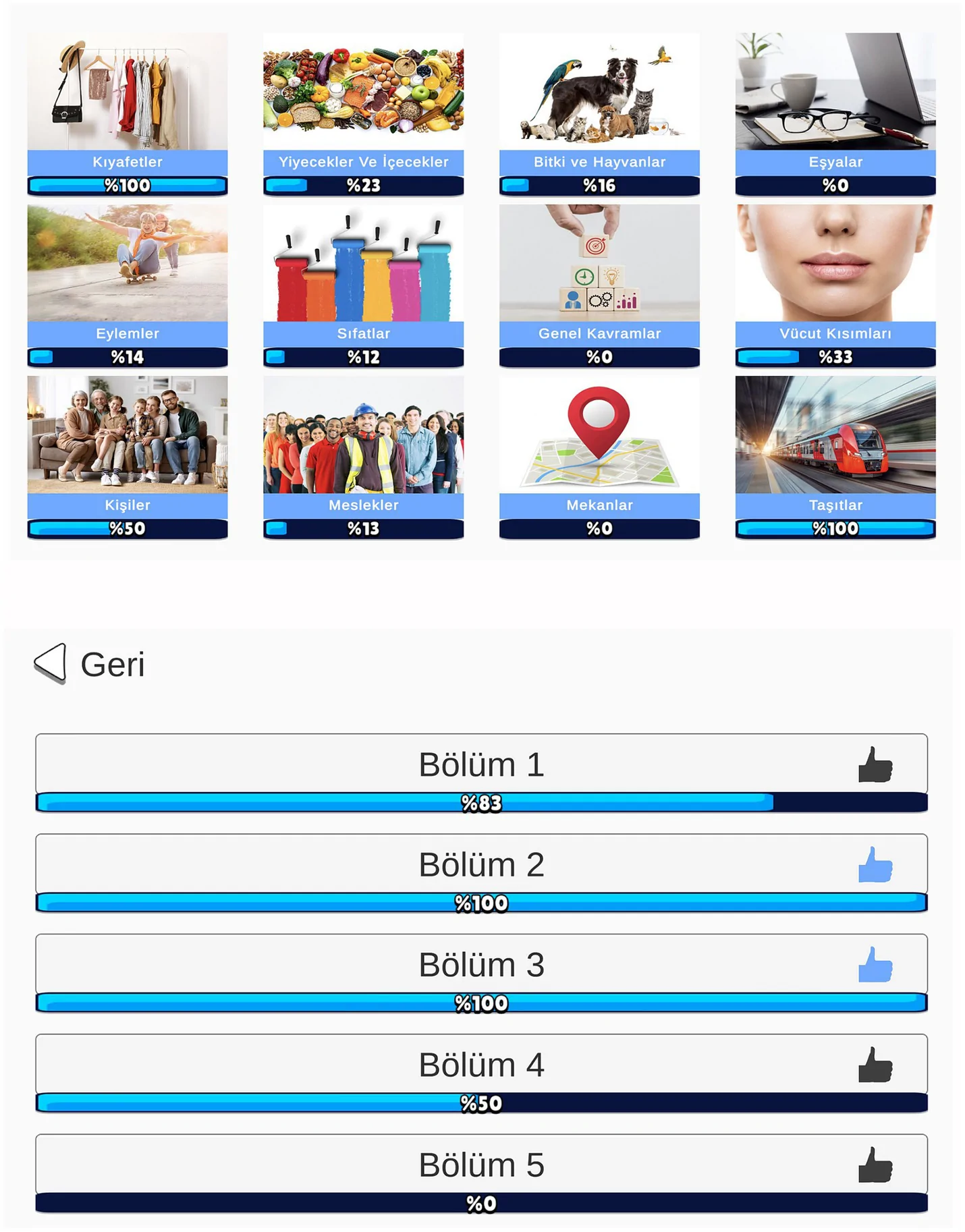
(From top to bottom) Progress bars showing completion in categories and sections. Images adapted with permission from iStock (https://www.istockphoto.com).

##### Intended clinical use model

The application was designed for clinician-guided use with structured home-based practice, rather than as a stand-alone self-treatment tool. A speech and language therapist conducts the initial assessment, selects the semantic categories most relevant to the individual’s needs, and configures the task parameters described above according to aphasia type and severity.

Between clinic sessions, the patient continues home practice with caregiver support, while the application automatically records performance indicators (e.g., accuracy, response time, time spent within the application, completed sections, and progress data) within the device. These data can subsequently be reviewed by the clinician directly on the patient’s device during follow-up sessions to monitor progress and adjust therapy parameters as needed. This device-based review model is consistent with the current preclinical stage of the application; remote data synchronization and clinician dashboards are planned for future versions.

This hybrid model aims to extend therapy intensity beyond in-person sessions while maintaining professional oversight. In contexts where access to specialist services is limited by geographic or economic barriers, the application may serve as a supplementary resource; however, integration with clinician-led therapy remains the primary intended model.

#### Evaluation of the mobile application’s usability

The mobile application was developed for the Android operating system and evaluated on standard consumer smartphones. Usability testing was conducted using touchscreen devices with screen sizes ranging from approximately 5.5 to 6.5 inches, reflecting commonly used smartphone displays at the time of data collection. All testing sessions were conducted under supervised conditions.

The usability evaluation of the mobile application included 40 individuals (20 female, 20 male) with normal hearing and Mini-Mental State Examination scores of 24 or above. Participants were divided into three age groups: 18–39 years (*n* = 15), 40–59 years (*n* = 12), and 60 years and above (*n* = 13). Participants tested the application prototype and were instructed to complete sections across the 12 categories. All trials were conducted at the “hard” difficulty level with six options. Following the instruction “Select the image representing the word you hear as quickly as possible by tapping on it,” participants were asked to proceed without taking breaks during the section. This allowed calculation of both the total duration for each section and the average time per question. In all other cases, participants were informed they could pause the application at any time.

A practice session was conducted prior to testing. To minimize learning effects, participants were asked to complete each section in a single attempt. The percentage of correct responses (PCR) and response time (RT) for all categories were recorded for analysis. PCR was calculated based on the participant’s first response to each item. Responses were scored as correct or incorrect based on the initial selection only; repeated attempts following an incorrect response did not alter the score, as these subsequent steps were intended for therapeutic purposes. Response time was defined as the total duration from the presentation of the auditory stimulus to the participant’s final selection, rather than pure reaction time, due to the interactive nature of the task and the possibility of repeated listening or cue use. Based on feedback collected through the MAUS, the application was revised to address aspects participants identified as unclear or impractical.

### Data analysis

Data were analyzed with IBM SPSS Statistics 20.0. Descriptive statistics (mean, SD, median) were reported. Relationships between image agreement, name agreement (%), and *H* values were examined using Spearman’s and Pearson’s correlations. The Kruskal–Wallis test was employed for score and time comparisons across age groups (by category and overall) and to assess associations with education level and daily mobile device use. Skills-based score/time differences were evaluated using the Mann–Whitney *U* test. Significance was set at *p* < 0.05.

#### Handling of response time outliers

Because response time is continuous and potentially sensitive to extreme values, an outlier analysis was conducted for RT data. Response time values exceeding ±3 SD from each participant’s category-specific mean were identified as outliers (0.9% of trials excluded). Reanalysis of the trimmed dataset showed that the pattern and direction of all group differences remained consistent, indicating that the findings were not driven by extreme response time values potentially related to participant distraction. Primary analyses were therefore conducted on the full dataset.

## Results

All results presented below are based on data obtained from healthy adult participants and reflect preliminary performance and usability characteristics of the mobile application.

### Descriptive statistics by demographic variables

To evaluate the usability of the mobile application, the 18–39, 40–59, and ≥ 60 age groups were compared with respect to sex, educational status, mobile device use skills, and daily mobile device use. Chi-square tests were used to compare sex distributions across groups, and no significant difference was observed (*p* > 0.05). Educational status was similarly compared across groups; no significant differences were found in the proportions with primary, secondary, undergraduate, or postgraduate education (*p* > 0.05).

Data on mobile device use collected via the participant information form were analyzed using the chi-square test. A statistically significant association was found between mobile device use skills and age group (*p* < 0.01). There was also a significant difference among groups in daily mobile device use (*p* < 0.05).

### Evaluation of the images used in the mobile application

To standardize the images used in the application, subjective measures were employed to determine name agreement and image agreement values. The name agreement study included 41 participants. For each image, name agreement (%)—the percentage of the most frequently produced name—and the H value—computed from the proportions of all distinct responses—were calculated.

The image agreement study included 43 participants. After mentally visualizing the given word, participants rated, on a five-point Likert scale, how closely the presented image matched the mental image they had formed. Scores for each word were recorded and mean values were calculated. Table 1 presents the mean, standard deviation, maximum, and minimum for image agreement and for name agreement (percentage and *H* value).

**Table 1:** Image agreement, name agreement (%), and name agreement (*H*) values for the images used in the mobile application.

Variables	Mean	SD	Median	25%	75%	Min	Max
Name agreement (%)	87.23	16.33	95.00	80.00	100.00	40.00	100.00
Name agreement (H)	0.51	0.58	0.29	0.00	0.85	0.00	2.88
Image agreement	4.48	0.39	4.50	4.28	4.80	1.50	5.00

The relationships among name agreement (%), name agreement *H* value, and image agreement were evaluated using Spearman’s rank-order correlation (Spearman’s rho). A strong negative correlation was found between name agreement (%) and the *H* value, a moderate negative correlation between image agreement and the *H* value, and a moderate positive correlation between name agreement (%) and image agreement (Table 2). In other words, as expected, increases in name agreement (%) and image agreement were associated with decreases in the *H* value (and vice versa).

**Table 2:** Correlation analysis of image agreement, name agreement (%), and name agreement (*H*) values.

Variables	Name agreement (%)	Name agreement (*H*)	Image agreement
Name agreement (%)	1.00	**−0.982** ^**a**^	**0.327** ^**a**^
Name agreement (H)	**−0.982** ^**a**^	1.00	**−0.337** ^**a**^
Image agreement	**0.327** ^**a**^	**−0.337** ^**a**^	1.00

a Correlation is significant at the 0.01 level (two-tailed). Bold values indicate statistical significance.

As a result of these analyses, images with an *H* value ≥1 were replaced with alternatives judged to represent the target word more accurately. For example, for the target word “different,” the initial image yielded *H* = 1.98. Because the second most frequent response was “long,” the image was considered misleading and was replaced (Figure 6).

**Figure 6:**
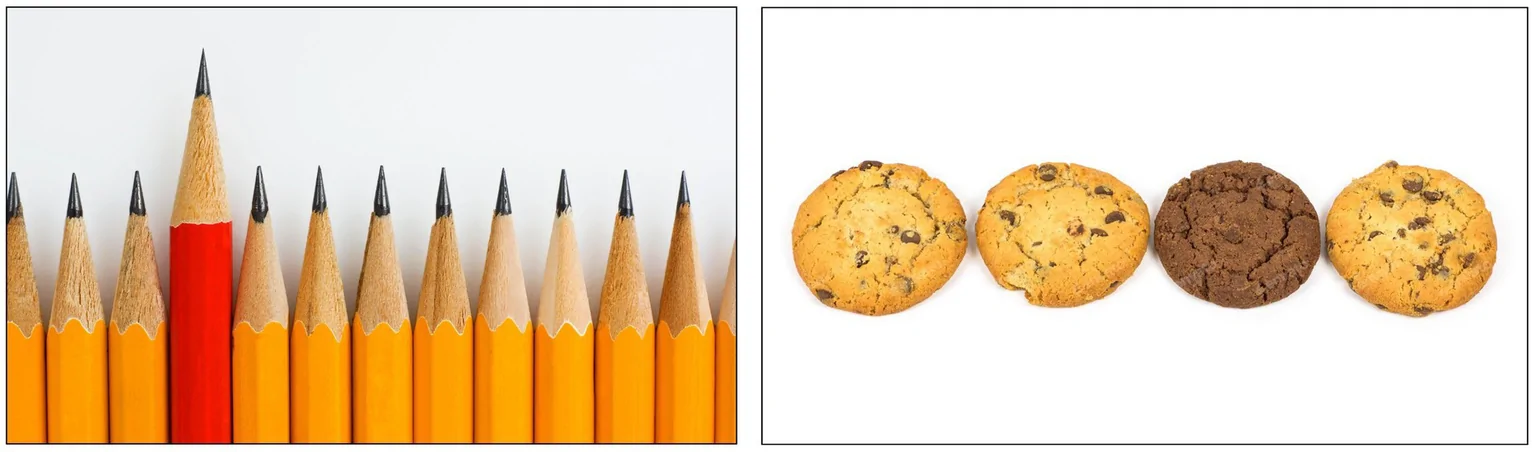
Initial and final images selected for the target word “different.” The image on the left shows the first image chosen for the target word “different,” while the image on the right shows the second image that replaced the first due to its high *H* value.

### Evaluation of user performance in terms of percentage of correct responses and response time

Comparisons of PCR and RT across age groups were performed using the Kruskal–Wallis test. When significant differences were detected, the Mann–Whitney *U* test was applied to determine which age groups differed.

Table 3 presents the results of PCR comparisons for each category across age groups. According to the Kruskal–Wallis test, the categories “Verbs,” “Adjectives,” and “General Concepts” showed statistically significant differences in PCR between age groups (*p* < 0.05). Mean PCR across all categories (mPCR) also differed significantly between age groups (*p* < 0.05).

**Table 3:** Comparison of the percentage of correct responses (%) for each category according to age group.

Category	Age groups									*H* (df)	*p*
	18–39 years			40–59 years			60 years and above				
	Mean	SD	Median	Mean	SD	Median	Mean	SD	Median		
Clothing	97.33	3.25	97.75	96.33	2.92	97.00	96.04	3.70	95.75	1.544 (2)	0.462
Foods and drinks	98.53	1.50	99.07	96.55	2.58	96.46	96.35	3.53	96.84	3.617 (2)	0.164
Plants and animals	97.91	2.08	98.50	93.20	9.04	98.41	93.46	7.85	96.83	2.220 (2)	0.329
Objects	97.16	2.29	97.20	97.26	1.83	97.23	95.92	2.70	96.53	2.175 (2)	0.337
Verbs	97.28	2.20	97.92	93.96	5.09	94.92	92.67	3.81	93.46	11.327 (2)	**0.003**
Adjectives	97.90	1.69	97.75	94.13	5.05	94.31	91.08	5.26	91.37	12.717 (2)	**0.002**
General concepts	98.65	1.64	99.11	94.33	5.45	96.27	94.58	4.12	95.77	8.474 (2)	**0.014**
Body parts	96.93	3.41	99.66	94.66	4.27	94.33	92.92	6.26	94.00	3.850 (2)	0.146
People	95.00	5.00	95.00	96.81	3.21	95.90	93.07	7.22	95.00	1.527 (2)	0.466
Occupations	96.84	2.48	98.00	94.80	4.17	95.00	92.93	4.74	91.83	4.208 (2)	0.122
Places	95.20	3.50	95.71	91.88	6.74	92.65	93.39	5.56	93.57	1.210 (2)	0.546
Vehicles	99.00	2.07	100.00	97.27	3.27	98.63	98.84	2.19	100.00	3.157 (2)	0.206
mPCR	97.31	1.30	97.71	95.05	3.29	96.28	94.27	2.94	94.17	7.977 (2)	**0.019**

mPCR, mean percentage of correct responses across all categories. Bold values indicate statistical significance.

According to the Mann–Whitney *U* test, applied to categories identified as significantly different by the Kruskal–Wallis test, there was no significant difference in mPCR between the 18–39 and 40–59 age groups (*p* = 0.07). The 18–39 age group had a significantly higher mPCR than the 60+ group (*p* = 0.005). No significant difference was observed between the 40–59 and 60+ age groups (*p* = 0.514).

Across the three age groups, statistically significant differences were observed in RT for each of the 12 categories and for the mean RT across all categories (mRT). *Post hoc* Mann–Whitney *U* tests indicated that the 18–39 group had a lower mRT than the 40–59 group, although this was not statistically significant (*p* = 0.130). The 18–39 group showed a significantly lower mRT than the ≥60 group (*p* < 0.001). The 40–59 group also had a significantly lower mRT than the ≥60 group (*p* = 0.008) (Figure 7).

**Figure 7:**
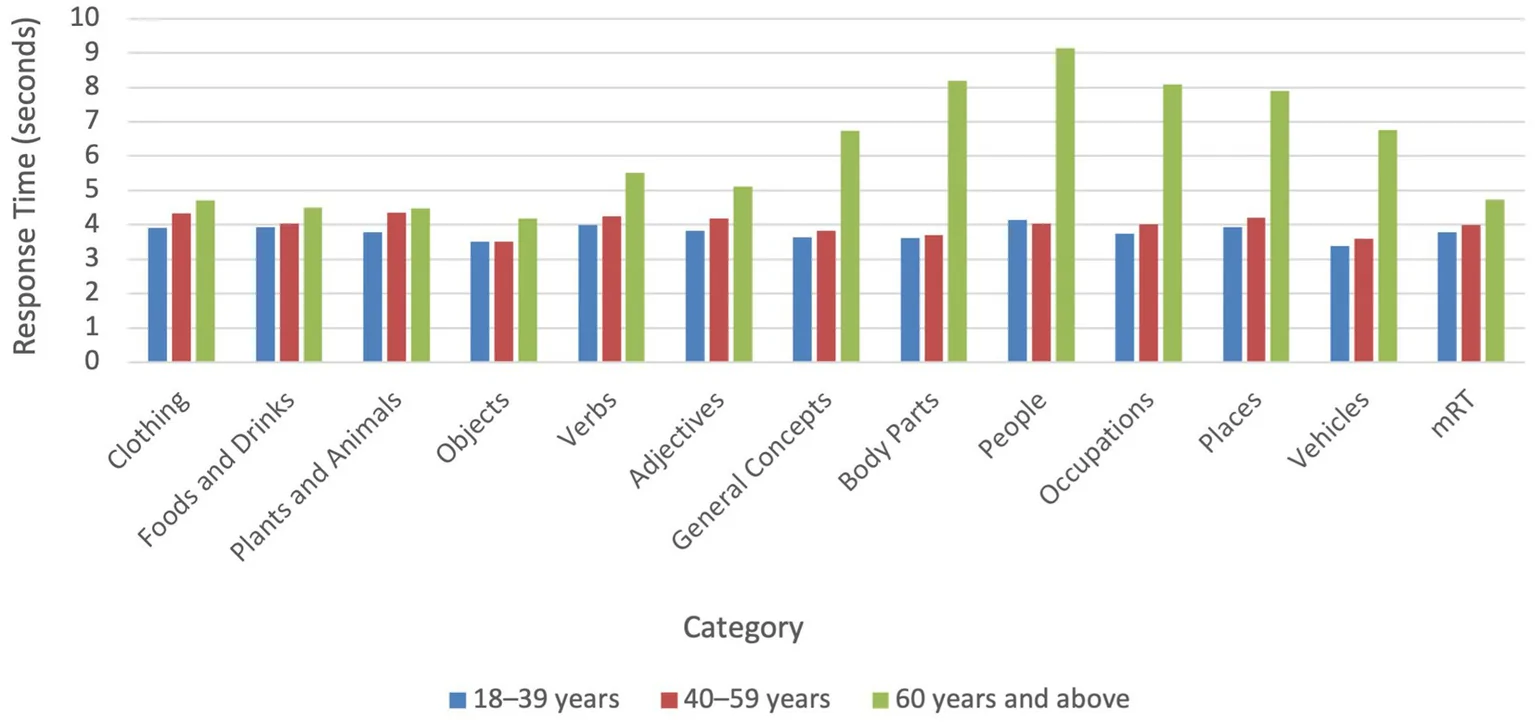
Response time (seconds) for each category by age group. mRT: mean response time across all categories.

Participants were grouped by education level as primary school, high school, undergraduate, or postgraduate. PCR and RT for each category were compared across education groups using the Kruskal–Wallis test. Where significant differences emerged, Mann–Whitney U tests identified the pairs that differed. Statistically significant differences were found between education levels in the “Adjectives,” “Vehicles,” and “Body Parts” categories (*p* < 0.05). mPCR also showed statistically significant differences between groups (*p* < 0.05). According to the Mann–Whitney U test, significant differences in mPCR were observed between primary school and undergraduate (*p* = 0.045), primary school and postgraduate (*p* = 0.011), high school and postgraduate (*p* = 0.039), and undergraduate and postgraduate (*p* = 0.014) education levels.

In RT comparisons using the Kruskal–Wallis test, significant differences emerged between education groups in the “Foods and Drinks,” “Objects,” “Adjectives,” “General Concepts,” “Body Parts,” “People,” “Occupations,” “Places,” and “Vehicles” categories (*p* < 0.05). The mRT also differed significantly between groups (*p* = 0.007) (Table 4). According to the Mann–Whitney U test, mRT differed significantly between primary school and undergraduate (*p* = 0.038), primary school and postgraduate (*p* = 0.011), and high school and postgraduate (*p* = 0.003).

**Table 4:** Comparison of response time (seconds) for each category according to education level.

Category	Education level												*H* (df)	*p*
	Primary school			High school			Undergraduate			Postgraduate				
	Mean	SD	Median	Mean	SD	Median	Mean	SD	Median	Mean	SD	Median		
Clothing	4.45	0.45	4.63	4.62	1.15	4.17	4.21	0.68	4.10	3.74	0.46	3.75	4.496 (3)	0.213
Foods and drinks	4.39	0.67	4.25	4.25	0.63	4.26	3.92	0.51	3.99	3.58	0.49	3.71	9.305 (3)	**0.026**
Plants and animals	4.32	0.73	4.22	4.13	0.72	3.92	4.33	1.45	4.01	3.34	0.55	3.24	6.305 (3)	0.098
Objects	4.13	0.64	4.00	3.81	0.40	3.73	3.74	0.60	3.77	3.21	0.29	3.17	9.458 (3)	**0.024**
Verbs	5.81	1.55	5.73	4.64	1.04	4.44	4.43	0.95	4.35	3.71	0.71	3.76	7.237 (3)	0.065
Adjectives	4.96	0.70	5.31	4.73	0.92	4.62	4.11	0.77	4.06	3.78	0.66	3.71	9.911 (3)	**0.019**
General concepts	4.96	0.79	4.63	4.08	0.62	4.24	3.85	0.48	3.83	3.49	0.37	3.66	11.29 (3)	**0.010**
Body parts	5.03	1.02	5.63	4.01	0.69	3.95	3.83	0.52	3.71	3.36	0.46	3.52	10.02 (3)	**0.018**
People	5.90	1.56	5.95	4.54	0.76	4.38	4.29	0.65	4.30	3.78	0.63	4.05	8.84 (3)	**0.031**
Occupations	5.25	0.78	5.73	4.34	0.57	4.24	4.01	0.52	3.92	3.54	0.56	3.69	11.969 (3)	**0.007**
Places	5.21	0.77	5.60	4.45	0.51	4.32	4.22	0.63	4.09	3.56	0.60	3.56	11.626 (3)	**0.009**
Vehicles	4.75	0.87	4.15	3.76	0.28	3.73	3.49	0.40	3.60	3.21	0.45	3.25	16.299 (3)	**<0.001**
mRT	4.93	0.81	4.97	4.34	0.50	4.25	4.03	0.54	4.11	3.52	0.42	3.61	12.048 (3)	**0.007**

mRT, mean response time across all categories. Bold values indicate statistical significance.

Based on the Participant Information Form, participants were categorized by mobile device use skills as “Poor,” “Moderate,” “Good,” and “Very Good.” For analysis, these were dichotomized into “Moderate and Below” and “Good and Above.” The Mann–Whitney *U* test was used for comparisons of PCR and RT in each category. PCR comparisons based on mobile device use skills revealed significant differences between the groups in the categories “Verbs,” “Adjectives,” and “Occupations” (*p* < 0.01) and “General Concepts” (*p* < 0.05). A significant difference was also observed in mPCR between the two groups (*p* < 0.01). Across these categories, participants with “Good and Above” mobile device use skills scored higher than those with “Moderate and Below” skills (Figure 8). Significant differences in RT were found between the two groups in the categories “Verbs,” “Adjectives,” “General Concepts,” and “Occupations.” mRT also differed significantly between the groups (*p* < 0.01), with the “Moderate and Below” group showing longer response time than the “Good and Above” group.

**Figure 8:**
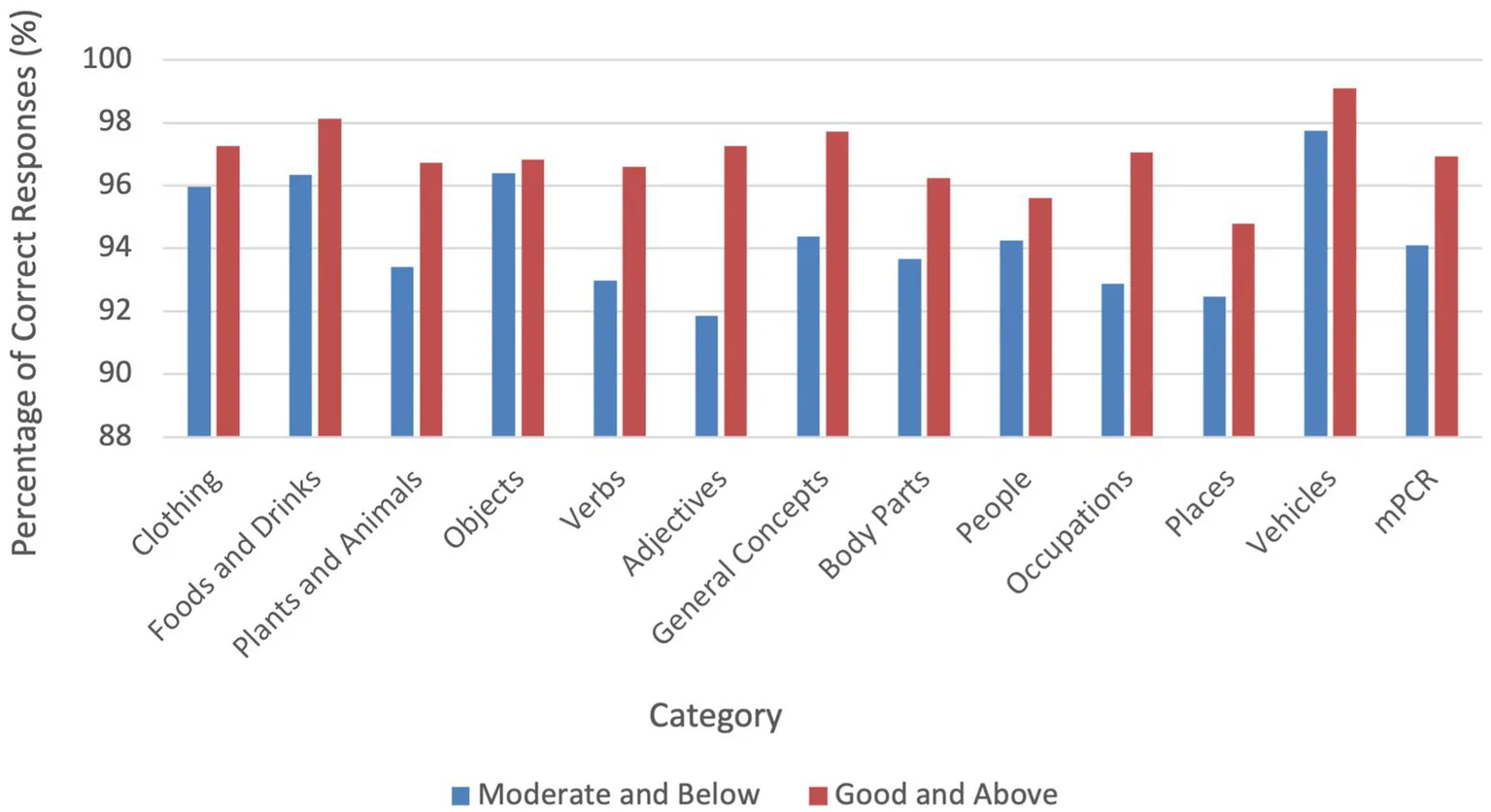
Percentage of correct responses (%) across categories by mobile device use skills. mPCR: mean percentage of correct responses across all categories.

Participants were grouped by daily mobile device use into categories of 0–2 h, 2–4 h, and ≥5 h. Group comparisons were conducted using the Kruskal–Wallis test, with significant results further analyzed by the Mann–Whitney U test. No category showed a significant difference in PCR across the three groups (*p* > 0.05). For RT, a significant difference emerged only in Foods and Drinks. Pairwise tests indicated that the 0–2 h group had longer RT than the ≥5 h group in this category (*p* = 0.010); 0–2 h vs. 2–4 h (*p* = 0.061) and 2–4 h vs. ≥ 5 h (*p* = 0.472) were not significant. Nevertheless, the mean mRT tended to be highest in the 0–2 h group, lower in the 2–4 h group, and lowest in the ≥ 5 h group.

### Analysis of MAUS results

After completing the tasks, participants filled out the Mobile Application Usability Scale (MAUS). MAUS results were calculated as the mean scores of the subscales—Aesthetic, Color, Entry, Control, Fingertip, Font, Gestalt, Hierarchy, Animation, and Transition—as well as the total score. Descriptive statistics (mean, standard deviation, median, minimum, maximum) for MAUS subscales and the total score are presented in Table 5. The following ranges were used to interpret the arithmetic means of MAUS ratings: 1.00–1.86 = definitely not usable, 1.87–2.72 = not usable, 2.73–3.58 = partly not usable, 3.59–4.44 = undecided, 4.45–5.30 = partly usable, 5.31–6.16 = usable, 6.17–7.00 = definitely usable.

**Table 5:** Descriptive statistics of MAUS subscales and total MAUS scores.

MAUS subscales	Mean	SD	Median	Min	Max
Aesthetic	6.03	0.92	6.00	3.00	7.00
Color	6.38	1.00	7.00	3.00	7.00
Entry	6.40	0.78	7.00	5.00	7.00
Control	6.50	0.72	7.00	5.00	7.00
Fingertip	6.23	1.23	7.00	2.00	7.00
Font	6.43	0.96	7.00	3.00	7.00
Gestalt	6.58	0.64	7.00	5.00	7.00
Hierarchy	6.68	0.57	7.00	5.00	7.00
Animation	5.98	1.40	6.50	1.00	7.00
Transition	6.00	1.06	6.00	3.00	7.00
MAUS total	6.32	0.62	6.60	4.30	7.00

Participants rated Color, Entry, Control, Fingertip, Font, Gestalt, and Hierarchy as “definitely usable”; Aesthetic, Animation, and Transition were rated “usable.” The lowest subscale mean was Animation (*M* = 5.98 ± 1.40), and the highest was Hierarchy (*M* = 6.68 ± 0.57). The MAUS total score averaged 6.32 ± 0.62, indicating that, overall, participants rated the mobile application as definitely usable.

## Discussion

Aphasia is a disorder resulting from brain damage that affects language functions such as speaking, comprehension, reading, and writing (53). Its most common cause is stroke, and it occurs in 20–38% of individuals after acute stroke (54, 55). Although auditory comprehension deficits in aphasia are among the symptoms causing the greatest communication restrictions, they have received insufficient attention in the literature (5).

The American Heart Association recommends 5 days of post-stroke rehabilitation per week, 3 h daily (56, 57). Research confirms that intensive therapy is more effective (58). However, most centers cannot provide therapy at this intensity (59). In Türkiye, access is even more limited, particularly for individuals outside major cities, due to the lack of facilities and personnel. This underscores the importance of technology-assisted interventions (9, 10).

A number of mobile- and tablet-based applications co-designed with people with aphasia have been developed in English-speaking countries, including Constant Therapy (16), TactusTherapy (19), PhotoTalk (20), RecoverNow (21, 25), MakeWrite (23), StepByStep Therapy (24), and BangaSpeak (22) [see Griffel et al. (37) for an overview]. Recent systematic reviews in this field consistently report measurable gains in language performance with mobile- and tablet-based interventions, although effect sizes vary substantially across linguistic domains and study designs (17, 18). Yet, this growing international literature contrasts sharply with the scarcity of comparable resources for Turkish-speaking individuals. Currently, only one application in Türkiye (26) targets naming skills, with none addressing auditory comprehension. To bridge this gap, the present study developed a mobile application for auditory comprehension in aphasia through three stages: preliminary preparation (objectives, content, materials), name and image agreement testing of visual stimuli, and user experience analysis with MAUS.

### Selection of words used in the mobile application

In assessment batteries and therapy programs targeting language comprehension and production, the control of psycholinguistic parameters is essential (60–62). In our application, exercises were hierarchically structured according to familiarity, imageability, and age of acquisition. The influence of these variables and word frequency on processing and comprehension has been highlighted in the literature (61–63). Therefore, 1,128 words (nouns, adjectives, and verbs), determined from frequency lists with linguistic support and selected based on familiarity, imageability, and age of acquisition values previously documented in Turkish research (39), were incorporated into the application.

### Evaluation of the images used in the mobile application

From the 1,128 words analyzed in the previous stage, 778 with high imageability were selected, and appropriate visual stimuli were identified. The present study, conducted with 84 participants using these visuals, revealed a strong negative correlation between naming agreement (%) and *H* value (rs = −0.982), a moderate positive correlation between name agreement (%) and image agreement (rs = 0.327), and a moderate negative correlation between *H* value and image agreement (rs = −0.337). In other words, as the *H* value increases, both image agreement and name agreement (%) decrease. Similar results have also been reported by Ghasisin et al. (64) and Atalık (26).

Snodgrass and Vanderwart (40) images have been standardized in several languages [English: Barry et al. (60); French: Alario and Ferrand (65); Italian: Nisi and Longoni (66); Japanese: Nishimoto et al. (67)]. Across these studies, high negative correlations between name agreement (%) and *H* value, weak-to-moderate positive correlations between name agreement (%) and image agreement, and weak-to-moderate negative correlations between *H* value and image agreement have been observed. The results of our study are consistent with the literature. A low *H* value indicates high naming agreement, whereas images with high image agreement tend to generate clearer associations. Conversely, low image agreement is associated with increased activation of alternative lexical items.

### Development of the mobile application for auditory comprehension skills

The application is based on an auditory word-to-picture matching task, a paradigm grounded in cognitive neuropsychological models of single-word processing (68). Successful auditory comprehension in these models requires three components: the phonological input lexicon, the semantic system, and the mapping between them. Word-to-picture matching engages all three, since the listener must recognize the spoken word, access its meaning, and match it to a picture (68, 69). Repeated practice on this task is expected to strengthen lexical–semantic processing, in line with established principles of impairment-based aphasia therapy (69, 70). The application is therefore intended for individuals with auditory comprehension deficits affecting either the phonological input lexicon or the semantic system, although the mechanism of improvement may differ across these profiles (68). The configurable distractor settings further allow the task to be tailored to the patient’s profile: semantically related distractors place greater demand on semantic discrimination, while unrelated distractors mainly test word-form recognition.

The 12 semantic categories in the mobile application were drawn from studies of semantic fluency (41–46) and semantically based aphasia interventions (47, 71). Each category comprises sections ordered by difficulty level (sections were determined based on the psycholinguistic properties of the words). Drawing upon foundational studies by Brookshire and colleagues (48–52), the hierarchical order of difficulty was designed in line with more recent evidence that graded task difficulty and errorless learning principles support performance and engagement in aphasia rehabilitation (72). Brookshire (49) emphasized that arranging tasks from easy to difficult increases the success of individuals with aphasia and that a low error rate supports motivation. Since presenting difficult tasks first may lead to failure, a progression from easy to difficult is recommended.

The “Settings” section of the application includes three difficulty levels—easy, medium, and hard—determined by the semantic relatedness between the target and distractors. Duffy and Watkins (73) reported that semantically unrelated distractors enhance performance, whereas Pizzamiglio and Appicciafuoco (74) emphasized that semantically related distractors increase task difficulty. Chieffi et al. (75) showed that semantic proximity is more influential than visual-perceptual similarity. In their study evaluating individuals with aphasia on a word comprehension task, participants made more errors when the response options were semantically related (e.g., banana, apple, grape) than when they were visually related (e.g., wheel, button, lifebuoy). Recent psycholinguistic work has further confirmed that semantic competition between target and distractors is a major source of difficulty in word comprehension and naming, with semantically related distractors slowing processing and increasing error rates in both healthy adults and individuals with aphasia (76, 77).

To support users who may struggle with auditory input alone, a “Hint” icon allows the written form of the target word to be displayed on demand, providing an additional orthographic cue as needed. This optional integration of written input alongside the auditory stimulus and the visual array reflects multimodal principles in aphasia intervention, where combining auditory, visual, and written input has been shown to enhance lexical retrieval and engagement (52, 78–83). Goodglass et al. (80) reported that visual stimuli elicited the fastest responses, while Moore (81) showed that multimodal stimuli support left hemisphere activation.

After each incorrect response, the auditory stimulus is replayed and can also be listened to again by selecting the “speaker” icon in the application. Repetitive sensory stimulation is consistent with the therapeutic approaches of Schuell (78). LaPointe et al. (84) demonstrated that repeating commands in the Token Test increased auditory comprehension in individuals with aphasia. Consistent with these earlier findings, contemporary models of aphasia rehabilitation continue to emphasize that repeated exposure and structured stimulation are key drivers of language recovery (7, 85, 86).

Feedback design plays a complementary role in auditory comprehension training. Schuell (78) argued that the initial stimulus is more effective than feedback, and that excessive feedback may overload the patient. Stoicheff (87) noted that statements such as “you are wrong” reduce motivation, while Holland and Sonderman (88) reported that excessive explanations during auditory comprehension tasks may cause confusion. Building on these findings, the application provides feedback only through non-verbal stereotypical sounds after correct or incorrect responses, thereby avoiding explanatory statements. This minimal-feedback design is aligned with errorless and low-error learning principles in modern aphasia rehabilitation, which suggest that reducing reliance on explicit corrective feedback can support performance and reduce frustration during practice (72). In addition, progress bars visually display the user’s improvement over time. Brookshire (89) reported that visual presentation of progress reinforces behavior and supports therapeutic engagement. More recent work on technology-supported aphasia therapy similarly highlights that visual feedback on progress enhances user engagement and adherence during home-based practice (13, 90).

### Evaluation of user performance in terms of percentage of correct responses and response time

The developed application was used by 40 participants divided into age groups (18–39, 40–59, 60+), and their percentage of correct responses (PCR) and response time (RT) were analyzed. Significant differences were found between age groups in the PCRs of the “Verbs,” “Adjectives,” and “General Concepts” categories, as well as in the mPCR (*p* < 0.05). The mPCR of the 18–39 age group was significantly higher than that of participants aged 60 and above (*p* = 0.005). In terms of RT, there was a significant difference among all age groups; RT increased with age (*p* < 0.05). As age increases, comfort with using mobile applications decreases. It is known that mobile health applications are mostly used by individuals under the age of 35, and that a portion of individuals over the age of 60 have no prior experience with mobile applications (91, 92). In their review addressing potential problems in mobile health application use with aging, Wildenbos et al. (93) stated that older adults’ cognitive, physical, and perceptual abilities, as well as motivational states may negatively affect their mobile health experiences. This finding is consistent with the lower scores and longer RT observed in the older age groups in the present study.

When participants were grouped according to educational level, significant differences were found in the PCRs of the “Adjectives,” “Vehicles,” and “Body Parts” categories, as well as in the mPCR (*p* < 0.05). The primary school group scored significantly lower than the undergraduate and postgraduate groups; the high school group, significantly lower than the postgraduate group; and the undergraduate group, significantly lower than the postgraduate group (*p* < 0.05). In addition, the primary school group had significantly longer RT compared to the undergraduate and postgraduate groups, and the high school group, significantly longer RT compared to the postgraduate group (*p* < 0.05). According to Krebs and Duncan (94), individuals with higher education levels tend to use mobile health applications more regularly. Therefore, it can be expected that as education level increases, familiarity with the application and performance also improve.

Participants whose mobile device use skills were rated as “Good and Above” performed better in terms of mPCR and the PCRs of the “Verbs,” “Adjectives,” “Occupations,” and “General Concepts” categories, and their RT in these areas was also significantly shorter (*p* < 0.05). These individuals may have achieved higher accuracy and speed because they could use mobile device functions more easily and quickly. The differences were particularly evident in categories such as “Verbs,” “Adjectives,” “General Concepts,” and “Occupations,” which are more challenging to visualize. In these categories, indirect representations were used more frequently. For example, the adjective “poor” was represented by an image of a man with his empty pockets turned inside out. Experience with mobile applications likely facilitated and accelerated the understanding of such images.

Analysis of daily mobile device use revealed no significant overall differences between groups. The only significant difference was found in the “Foods and Drinks” category, where the RT of the group with 0–2 h of daily usage was significantly longer than that of those using their devices for 5 h or more (*p* < 0.05). In addition, the average mRT was highest in the 0–2 h group and lowest in the 5 + hour group. This suggests that time spent with the device may positively affect speed but does not result in a significant difference in accuracy.

Based on these results, age, educational level, and mobile device use skills appear to influence PCR and RT variables in our application, as anticipated. However, the PCR in any category and the mPCR did not fall below 90% in any group, supporting the use of 90% as the threshold for section completion. Furthermore, no participant reported difficulty using the application, indicating that the application could be used comfortably across all these groups.

### Analysis of the Mobile Application Usability Scale (MAUS) results

As mobile technologies have become more prevalent in daily life, the number of mobile application development initiatives has increased (12). However, not every application meets user expectations; its success is largely determined by the user experience (95). Usability is defined as the degree to which a system can be used by specified users to achieve specified goals effectively, efficiently, and satisfactorily (30). Users generally tend to prefer applications that are easy to use (96).

In this study, usability was evaluated using the Mobile Application Usability Scale (MAUS), developed by Hoehle et al. (35) and adapted into Turkish by Güler (36). Participants rated the subscales “Color,” “Entry,” “Control,” “Fingertip,” “Font,” “Gestalt,” and “Hierarchy” at the “definitely usable” level. This indicates that the application’s colors, entry menus, controls, font type/size, and arrangement of similar elements met their expectations. The highest score was obtained for the “Hierarchy” subscale (*M* = 6.68 ± 0.57), reflecting the application’s core organizational principle: a hierarchical structure where words are presented through categories and sections ordered by difficulty level. This finding aligns with the application’s design and indicates that participants found the hierarchical structure functional.

The “Aesthetic,” “Animation,” and “Transition” subscales were rated at the “usable” level. This may reflect some participants experiencing minor issues during screen transitions, as well as the lower prioritization of aesthetic design during development. Accordingly, the software was updated to improve transitions, and enhancing the aesthetic dimension was set as a future goal. Although “Animation” received the lowest score (*M* = 5.98 ± 1.40), the rating still falls within the “usable” range, indicating acceptable functionality, consistent with a design prioritizing operational efficiency.

Overall, participants rated the application as “definitely usable,” achieving a high average rating (*M* = 6.32 ± 0.62). Notably, no MAUS subscale scored below the “usable” threshold, indicating high overall user satisfaction.

### Comparison of the developed application with other mobile applications for individuals with aphasia

Increasing the frequency of language therapy supports daily life participation for individuals with aphasia (6); however, many do not have access to such opportunities. In recent years, therapeutic mobile applications have emerged to help bridge this gap (97). Comprehensive reviews indicate that mobile- and computer-based interventions can deliver therapy at higher intensity and lower cost than conventional face-to-face approaches, while enabling independent practice outside the clinic (17, 18, 98). Over the past decade, various applications have been developed for individuals with aphasia, such as Constant Therapy, iTSA (98), iAphasia (99), TalkPath (100), VRRS-Tablet (101), and iPractice (13, 90).

These applications typically target multiple language modalities simultaneously, including auditory comprehension, naming, reading, and writing. As Repetto et al. (18) point out, this multi-target design is characteristic of many currently available applications. The application developed in the present study differs from this trend by focusing solely on word-level auditory comprehension—a deliberate choice aimed at enabling deeper, more finely graded training within a single domain. Accordingly, it incorporates a large dataset of 778 words and corresponding images, organized into a detailed hierarchical system based on psycholinguistic properties: words with high familiarity, high imageability, and low age of acquisition are placed in easier sections, while more challenging items are introduced gradually. This hierarchical organization reflects an established principle in aphasia rehabilitation, as graded learning has been shown to provide therapeutic benefits (48–52).

Our application shares certain features with Tactus Therapy (19), such as categorical organization, customizable difficulty levels, and the provision of feedback. However, in Tactus Therapy, words within semantic categories are presented in random order, whereas in the present application, words are grouped into sections based on psycholinguistic characteristics, and these sections are sequenced according to difficulty. Each section contains a fixed set of words, with random ordering only within the section. The design also allows free movement between sections, facilitating individualized use, while progress bars support motivation and enable performance tracking. This structured yet flexible design reflects two key principles that have been increasingly emphasized in recent reviews of mobile-based aphasia interventions. The first is the systematic adjustment of task difficulty based on user performance, which allows the therapy to remain appropriately challenging as the individual progresses. The second is the integration of task-specific practice, repetition, and structured feedback. Together, these principles have been consistently associated with the greatest therapeutic benefits (17, 102).

The only Turkish application previously developed for individuals with aphasia is Atalık’s (26), focusing on naming skills. Similar to our application, it targets a single language modality and presents words in categories. However, there are substantial differences in the target skill (auditory comprehension vs. naming), the core structure (hierarchical organization vs. sequential cueing), the size of the word set (778 vs. 219 words), and the overall design.

This comparison highlights a clear gap in Turkish-language mobile applications for individuals with aphasia and suggests that our application may offer a valuable contribution to the field. As the first Turkish mobile program specifically designed to target auditory comprehension skills, it represents an important step toward addressing this need. The results obtained from healthy participants demonstrated usability across different demographic groups, and feedback measured with the MAUS usability scale reflected high satisfaction. Nevertheless, there remains a need for additional Turkish-language mobile applications targeting other language modalities (e.g., repetition, reading, writing) and for studies evaluating their effectiveness in both short- and long-term therapeutic contexts.

### Limitations and future directions

The present study has several limitations that should be acknowledged, each pointing to important directions for future research. First, the development and evaluation of the application followed a multi-stage process involving stimulus selection, psycholinguistic standardization, interface design, and usability testing. As each stage was designed to verify feasibility and refine the application prior to clinical deployment, the evaluation phase was conducted with healthy adult participants only. This staged approach is consistent with established frameworks for digital health intervention development, in which feasibility and usability are first established in non-clinical samples before progressing to patient populations (37). Nevertheless, the present findings cannot be directly generalized to individuals with aphasia, whose performance is shaped by additional linguistic, cognitive, motor, and attentional challenges that may affect both task engagement and outcomes. To address this limitation, planned clinical studies will evaluate the application’s usability, acceptability, and therapeutic outcomes in individuals with aphasia across different clinical subtypes and severity levels.

A related limitation concerns the stimulus validation procedure: name and image agreement ratings were collected only from young healthy adults. Because aphasia predominantly affects older adults following stroke, age-related differences in lexical familiarity and naming performance may have influenced these normative values. This demographic is known to exhibit heightened sensitivity to name agreement and frequency effects, with reduced accuracy and longer latencies for low-frequency and later-acquired items (76, 103). Ideally, such norms should be collected from age-matched samples. Importantly, the subsequent evaluation of the finalized application across a wider adult age range yielded consistently high accuracy, suggesting that the selected stimuli and task structure remained accessible for older participants as well.

Second, the application was intentionally designed to operate at the single-word level, raising the important question of whether gains in recognizing isolated words translate to the sentence- and discourse-level comprehension that everyday communication demands. The available evidence suggests this progression is not automatic. In a systematic review of post-stroke aphasia interventions, Schultz et al. (104) found that most studies (55%) targeted the word level, yet transfer to natural dialogue remained inconsistent—largely because only 27% of interventions explicitly incorporated components designed to promote it. Reviews of computer- and mobile-based therapies report a similar pattern: generalization to untrained words and real-world communication is seldom assessed, and the few studies that do so report mixed results (17, 18, 98). Several design features of the present application were intended to bridge this gap: vocabulary organized into everyday semantic categories, items hierarchically ordered to strengthen lexical-semantic representations, and a clinician-guided use model with structured home practice tailored to each individual’s needs. Still, single-word training represents only an initial step, and future versions will extend the application to sentence and discourse levels, with upcoming clinical trials assessing whether these foundational gains transfer to functional communication in daily life.

Third, aphasia is a multidimensional disorder affecting multiple language modalities, including naming, repetition, reading, and writing, whereas the present application focuses exclusively on auditory comprehension. This reflects a deliberate design choice to provide a focused, psycholinguistically controlled tool allowing in-depth training within a single modality. While many digital interventions in the literature have adopted a multimodal structure incorporating several language domains within a single platform (17, 18), a single-modality tool cannot address the full range of impairments experienced by individuals with aphasia. To extend the scope of the application, additional modality-specific tools targeting these respective domains are planned, each built on the same methodological foundation and extensive stimulus sets applied here. These modules will ultimately be integrated into a comprehensive and modular digital intervention framework for aphasia rehabilitation.

## Data Availability

The datasets generated and analyzed during the current study are available from the corresponding author upon reasonable request. The data are not publicly available due to privacy restrictions related to participant information.

## Author’s note

This article is derived from the doctoral dissertation titled “Development of a Mobile Application to Support Intervention in Individuals with Aphasia Who Have Impaired Auditory Comprehension” (“İşitsel anlama becerilerinde bozulma olan afazili yetişkin bireylerde müdahaleye destek mobil uygulama geliştirilmesi”) completed at the Institute of Health Sciences, Ankara University, Ankara, Türkiye.
